# Supplementary material for: Clinical course and risk factors for sleep disturbance in patients with ischemic stroke
Source: PLoS One. 2022 Nov 8;17(11):e0277309. doi: 10.1371/journal.pone.0277309 (PMC9642877; doi:10.1371/journal.pone.0277309)
Supplement: Attachment — Submitted filename: Response to Reviewer PONE-D.22-17272docx.docx [file pone.0277309.s001.docx]

Response to Reviewer PONE-D-22-17272

1 - There are some previous studies on the prevalence of insomnia after stroke. I suggest elaborating on the similarities and differences in methods between the present study and previous ones.

Response:

In introduction, we have added the sentence. “Most studies investigated prevalence of insomnia in patients with ischemic assessing sleep quality after stroke onset. Prospective data on sleep quality in ischemic stroke patients before stroke, acute stroke and subacute stroke are not available.” (Page 5 to page 6).

2 - Some background information should be better put in context. For example, the general discussion on the core and penumbra seems off topic. It would be better replaced by a discussion on the stroke locations that are more likely to lead to insomnia.

Response:

In introduction, we add the sentence “During the acute stroke period, two dysfunctional areas affect neurological function which may affect sleep quality in patients with ischemic stroke.” (Page 4).

“Whether the neurological impairment in acute stroke phase of stroke may increase risk of insomnia and whether insomnia in acute stroke period many improves after reperfusion in penumbra requires further investigation.” (Page 5).

3 - Some factors such as the stress of hospital admission and the acute treatment of stroke may have an impact on sleep habits. This point should be further elaborated.

Response:

In discussion, we add the sentence “The condition could be related to anxiety, stress and treatment in acute stroke period. Whether insomnia in acute period but not in subacute or chronic stroke period related to reperfusion in penumbra area need further investigation.” (Page 14).

4 - Authors relied on subjective patient-reported outcomes, in the absence of objective measurements such as actigraphy or polysomnography. This could be a limitation to discuss.

Response

In discussion, we have add the limitation

“The diagnosis of insomnia was based on the scores of patient self-reported scale and absence the measurement of actinography or polysomnography.” (Page 15).

5 - How did Authors deal with patients with cognitive or language dysfunction? Patients must have responded to questionnaires for this study. If Authors excluded from the study patients with language and/or cognitive dysfunction, this would have led to selection bias.

Response:

This is the limitation of the study, due to the study depend patient self-report their sleep quality, we have state the limitation in discussion section.

“we did not include patients with aphasia, a previous stroke history, mental impairment, depression, critical medical disease, and patients who did not agree to enroll in the study, which may have selection bias. However, we have do our best to include the patients who can correctly report their sleep condition.” (Page 15).

6 - Authors state that most cases of insomnia occurring during the acute phase of stroke tend to resolve within 3 months. For that reason, in the Authors' opinion, it is not recommended to provide sleep medication to patients with stroke in the acute phase. In my opinion, this is not a strong recommendation as some patients with stroke might need sleep medication in the acute phase even if sleep disturbances do not last long. I suggest articulating this point in a better fashion.

Response:

We have changed to “In most cases, patients with insomnia symptoms in the acute stroke period recovered 3 months after stroke onset. Hence, pharmacotherapy for insomnia in the acute stroke period should be adopted individually^34^.” (Page 14).

7 - I see from the Figure that most patients without insomnia who developed insomnia during the acute phase of stroke resolve their condition, while most patients with previous history of insomnia are likely to remain in their condition after stroke. This point should be better discussed as it has clinical relevance. It would mean that previous history of insomnia should be seeked in patients with stroke to plan adequate interventions in the long term.

Response:

In discussion, we add the sentences

“Approximately half of patients with insomnia before stroke onset experienced insomnia at 3 month after stroke onset. The result underscore the importance of obtaining information on sleep quality prior to stroke. For improving the quality of life of stroke patients, it is Important to obtain the information of sleep quality before stroke and carry individualized treatment.” (Page 13 to page 14).

8 - Did insomnia influence stroke outcomes in terms of survival and post-stroke disability? Reporting data on this aspect could identify an area of intervention that would lead to benefits in post-stroke recovery.

Response:

Because of the study was designed to investigate the prevalence of insomnia in stroke patients, we did not record the outcome of stroke patients. At present, we cannot report the relationship between insomnia and outcome of stroke. This will be the subject of our next research.
