# Supplementary material for: Clinical course and risk factors for sleep disturbance in patients with ischemic stroke
Source: PLoS One. 2022 Nov 8;17(11):e0277309. doi: 10.1371/journal.pone.0277309 (PMC9642877; doi:10.1371/journal.pone.0277309)
Supplement: Attachment — Submitted filename: Response to reviewer 1. PONE-R2docx.docx [file pone.0277309.s002.docx]

Response to reviewer 1

We thank reviewer for their detail review and very important comments. Base on reviewer’s comments, we have made necessary changed point by point.

1 - I understand that the "chronic stage" of stroke is 3 months. In my opinion, it would be better to replace "chronic stage" with a more precise time indication.

Response: For precise timing indication, we have used chronic stage replace subacute stroke stage.

2 - The lack of outcome data on stroke should be stated as a major limitation of the study.

Response: We have add the limitation in discussion. In page 15, limitation section, we	add the sentence “We did not evaluate the impact of insomnia on the outcome	of patients with stroke.”

3 - English style should be carefully revised as there are many grammatical imprecisions.

Response: The English style of manuscript have been edited again.
